# Mechanisms of SNP and melatonin effects on antioxidant and chlorophyll metabolism in postharvest okra

**DOI:** 10.1038/s41538-025-00553-1

**Published:** 2025-08-25

**Authors:** Xianjun Chen, Yao Jiang, Jianwei Zhang, Xiaocheng Liu, Lulu Wang, Jintong Zheng, Jiayu Zeng, Qin Yang, Yan Zhou

**Affiliations:** 1 Provincial famous teacher Yang Qin studio/Key Laboratory of Molecular Breeding and Variety Creation of Horticultural Plants for Mountain Features in Guizhou Province, School of Life and Health Science, Kaili University, Kaili, China; 2 Life Science and Technology School, Lingnan Normal University, Zhanjiang, China; 3 GuangDong Technology Innovation Center of Tropical Characteristic Plant Resource Development, Lingnan Normal University, Zhanjiang, China; 4 Zhanjiang Key Laboratory of Tropical Characteristic Plant Technology Development, Lingnan Normal University, Zhanjiang, China

**Keywords:** Biochemistry, Plant sciences

## Abstract

Okra fruit rapidly undergoes chemical deterioration after harvest, necessitating effective preservation strategies. This study represents the first comprehensive investigation combining transcriptomic analysis with biochemical assessment to elucidate how SNPs and MT affect antioxidant capacity and chlorophyll metabolism in okra. MT + SNP treatment most effectively preserved fruit quality by reducing weight loss, maintaining color parameters, decreasing oxidative stress markers (H_2_O_2_, MDA), and enhancing antioxidant enzyme activities (SOD, CAT, POD, APX). This treatment stabilized chlorophyll content by modulating degrading enzymes (CLH, PPH, MDcase). Transcriptome analysis revealed 1927 differentially expressed genes associated with antioxidant defense and chlorophyll metabolism. Key antioxidant genes (*PODs*, *GSTs*, *MDHARs*) were upregulated, while chlorophyll metabolism genes (*POR*, *PAO*, *Lhcbs*) showed coordinated expression, maintaining pigment stability. Network analysis identified transcription factors (*NAC86*, *ERF4*, *MYB24*) linking these pathways. This study provides molecular insights for developing postharvest technologies that extend shelf life while maintaining nutritional quality and reducing food waste.

## Introduction

Okra (*Abelmoschus esculentus* L.) is a widely cultivated vegetable in tropical and subtropical regions, valued for its distinctive specific phytochemicals including dietary fiber (primarily pectins and hemicelluloses), phenolic compounds (quercetin derivatives and hydroxycinnamic acids), vitamins (A, B complex, C, E, and K), and essential minerals (Ca, K, Mg)^1^. The fruit also contains bioactive compounds with antioxidant, anti-inflammatory, and anti-diabetic properties, notably flavonoids, isoquercitrin, and unique mucilaginous polysaccharides that contribute to its health-promoting effects^1^. Despite these nutritional and functional attributes, postharvest okra fruit have a short shelf life and undergo rapid quality deterioration characterized by biochemical changes including chlorophyll degradation, cell wall polysaccharide breakdown, and oxidation of phenolic compounds^2^. These chemical transformations manifest as weight loss, color changes, and textural softening, substantially reducing consumer acceptability, nutritional value, and market potential. Various postharvest strategies have been investigated to maintain the stability of produce, including biological methods employing antagonistic microorganisms or natural compounds^3^, physical techniques such as low-temperature storage and modified atmosphere packaging^2^, and chemical approaches using phytohormones and antioxidants^4^.

Nevertheless, there remains a need for alternative or synergistic chemical interventions that can more effectively maintain the molecular integrity of postharvest produce. Postharvest deterioration in horticultural commodities is intricately linked to oxidative stress, primarily driven by the excessive accumulation of reactive oxygen species (ROS)^5^. At the molecular level, ROS cause lipid peroxidation of membrane phospholipids, protein oxidation, and DNA damage, resulting in disrupted cellular compartmentalization, increased membrane permeability, and compromised metabolic regulation^6^. Plants have evolved a sophisticated antioxidant system, encompassing both enzymatic [superoxide dismutase (SOD), catalase (CAT), peroxidase (POD), ascorbate peroxidase (APX)] and non-enzymatic defenses (ascorbic acid, glutathione, phenolic compounds), to neutralize ROS and maintain redox homeostasis^4^. Recent biochemical and molecular evidence has demonstrated that antioxidant-related genes not only scavenge excess ROS but also modulate key aspects of fruit ripening and quality retention through complex signaling networks^4,7^. For instance, overexpression of *SOD*, *CAT*, or *APX* in tomato and apple has been reported to inhibit ethylene biosynthesis and cell wall-degrading enzymes, thereby preserving fruit firmness and extending shelf life^7,8^. Similarly, enhanced activity of SOD, CAT, APX, and MDHAR in horticultural products was correlated with delayed softening and reduced decay incidence by maintaining membrane integrity and limiting oxidative damage to structural polysaccharides^4^. The efficacy of these defense networks during postharvest storage can be enhanced by exogenous signaling molecules, such as nitric oxide (NO) donors and melatonin (MT), both of which have been reported to improve antioxidant capacity and delay senescence in various perishable horticultural crops^9^. Notably, NO (frequently supplied via sodium nitroprusside, SNP) alleviates oxidative stress through direct chemical quenching of ROS and by inducing antioxidant enzymes, whereas melatonin functions as a potent antioxidant while also maintaining membrane phospholipid integrity, modulating gene expression, and preserving important physiological parameters^10^.

Chlorophyll metabolism represents another critical determinant of postharvest quality in green vegetables, with direct implications for visual appeal and nutritional value. Chlorophyll degradation leads to visible color shifts and potential loss of associated antioxidant compounds, often diminishing consumer acceptance and nutritional quality^11^. The chemical pathway of chlorophyll catabolism is orchestrated by several key enzymes, including chlorophyllase (CLH), which hydrolyzes the phytol chain; pheophytin pheophorbide hydrolase (PPH), which removes the central Mg^2+^ ion; and pheophorbide a oxygenase (PAO), which opens the tetrapyrrole ring^12–15^. Conversely, protochlorophyllide oxidoreductase (POR) and chlorophyllide a oxygenase (CAO) are pivotal for chlorophyll biosynthesis and interconversion between chlorophyll a and b^16^. Recent molecular research highlights the importance of transcription factors (TFs) that directly regulate these enzymes during postharvest senescence^11,17^. For instance, AP2/ERF-, MYB-, and NAC-family TFs bind to specific promoter elements of chlorophyll catabolic genes, thereby orchestrating pigment breakdown and color evolution in various horticultural produce^18–20^. Moreover, crosstalk among different TF families can finely tune the biosynthesis and degradation pathways of chlorophyll, as evidenced in citrus, where MYB-based regulatory networks simultaneously manage anthocyanin accumulation and chlorophyll breakdown^21^. The interplay of these TFs underscores the complex regulation of chlorophyll metabolism and suggests that exogenous regulators, such as MT and NO, may delay color loss in green produce by modulating both chlorophyll-related enzymes and their upstream transcriptional activators^9,22,23^. However, investigations into how SNPs and MT might jointly influence the stability of chlorophyll and related pigments in postharvest okra fruit have been limited.

Accordingly, this study aimed to evaluate the efficacy of SNP, MT, and their combined application (MT + SNP) in preserving specific phytochemicals, including phenolic compounds, chlorophyll pigments, and antioxidant molecules, as well as postharvest quality of okra fruit, focusing on weight loss, color parameters, antioxidant capacity, and chlorophyll metabolism. Notably, this represents the first comprehensive investigation to combine SNP and MT treatments for okra preservation, pioneering the use of integrated transcriptomic and biochemical approaches to reveal synergistic molecular mechanisms previously unknown in this system. We employed a combination of targeted biochemical analyses and untargeted transcriptomic profiling to elucidate the underlying molecular mechanisms, with an emphasis on identifying differentially expressed genes (DEGs) involved in antioxidant defense, chlorophyll metabolism, transcription factor regulation, and protein kinase signaling. Our novel application of weighted gene co-expression network analysis (WGCNA) enables the first identification of transcription factors that connect antioxidant and chlorophyll metabolism pathways in postharvest okra. The findings from this work not only shed light on the biochemical and genetic basis of exogenous NO and MT action but also establish new molecular insights into the regulatory networks governing redox homeostasis and pigment metabolism integration. This integrated approach addresses the critical need for effective, sustainable postharvest technologies that preserve both the commercial quality and functional food properties of fresh produce while providing the first molecular framework for understanding combined SNP-MT mechanisms in horticultural systems.

## Results

### Effects of SNP and MT on the weight loss and color of okra fruit

**Fig. 1:**
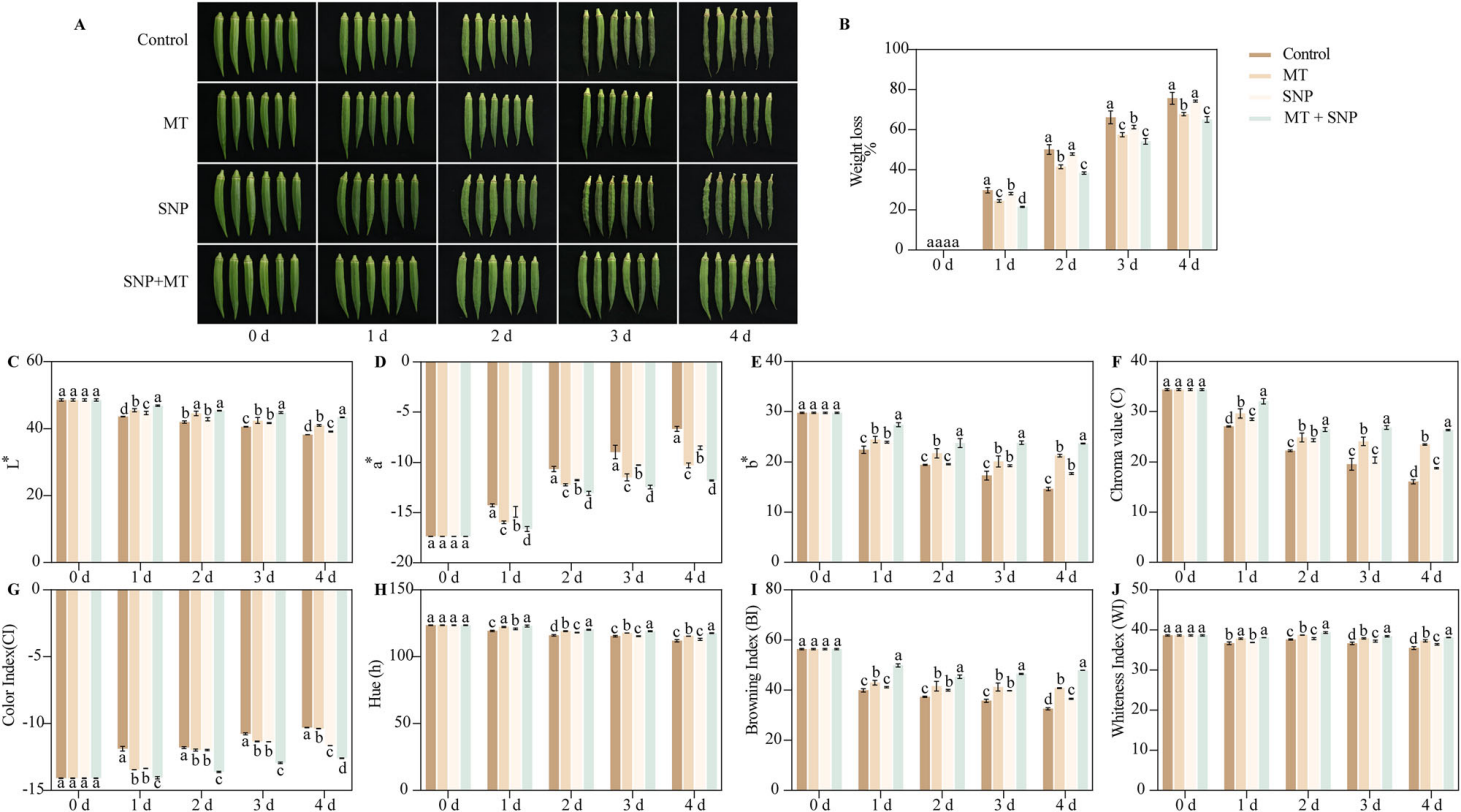
Effects of SNP and MT on quality-related indicators in okra fruit after 4 d of storage. Phenotype (**A**), weight loss (**B**), L* (lightness) value (**C**), a* (greenness; positive values) (**D**), b* (yellowness) (**E**), chroma (**F**), color index (positive values) (**G**), h (hueangle; positive values) (**H**), browning index (**I**), and whiteness index (**J**). Error bars represent SD (*n* = 3). Bars with different letters within a sampling date are significantly different (*P* < 0.05).

Postharvest okra fruit exhibited an increasing trend in weight loss during storage (Fig. 1B). Compared with the control, treatments with MT, SNP, and the combined MT + SNP resulted in lower weight loss rates throughout the storage period, except for the SNP treatment at 2 d and 4 d of storage. Notably, the MT + SNP treatment demonstrated the most effective mitigation, maintaining the lowest weight loss rate among all treatments. This combined intervention resulted in a reduction in weight loss of 14%–28% compared with the control group during the entire experimental period.

Over extended storage durations, the L^*^ values of all okra fruit displayed an increasing trend. Throughout the storage period, the MT, SNP, and MT + SNP treatments maintained lower L^*^ values compared with the control group (Fig. 1C). The MT + SNP treatment consistently yielded the lowest L^*^ values among all groups. The MT, SNP, and MT + SNP treatments reduced the a^*^ and CI values of okra fruit compared with the control throughout the entire storage period. Conversely, these treatments increased the b^*^ values (except for the SNP treatment at 2 d), chroma values (except for the SNP treatment at 3 d), *h* values (except for the SNP treatment at 3 d and 4 d), BI (except for the SNP treatment at 1 d), and WI values (except for the MT and MT + SNP treatments at 1 d, and the SNP treatment at 2 d) relative to the control. Notably, the MT + SNP treatment demonstrated enhanced effects by increasing the L^*^ values by 8%–13%, b^*^ values by 22%–62%, chroma values by 18%–64%, *h* values by 3%–5%, and BI by 21%–47%, while decreasing the a^*^ values by 17%–78% and CI values by 15%–22%, respectively, compared with the control throughout the storage period (Fig. 1D–J).

### Effects of SNP and MT on soluble protein, MDA, H_2_O_2_ content, and total antioxidant capacity in okra fruit

**Fig. 2:**
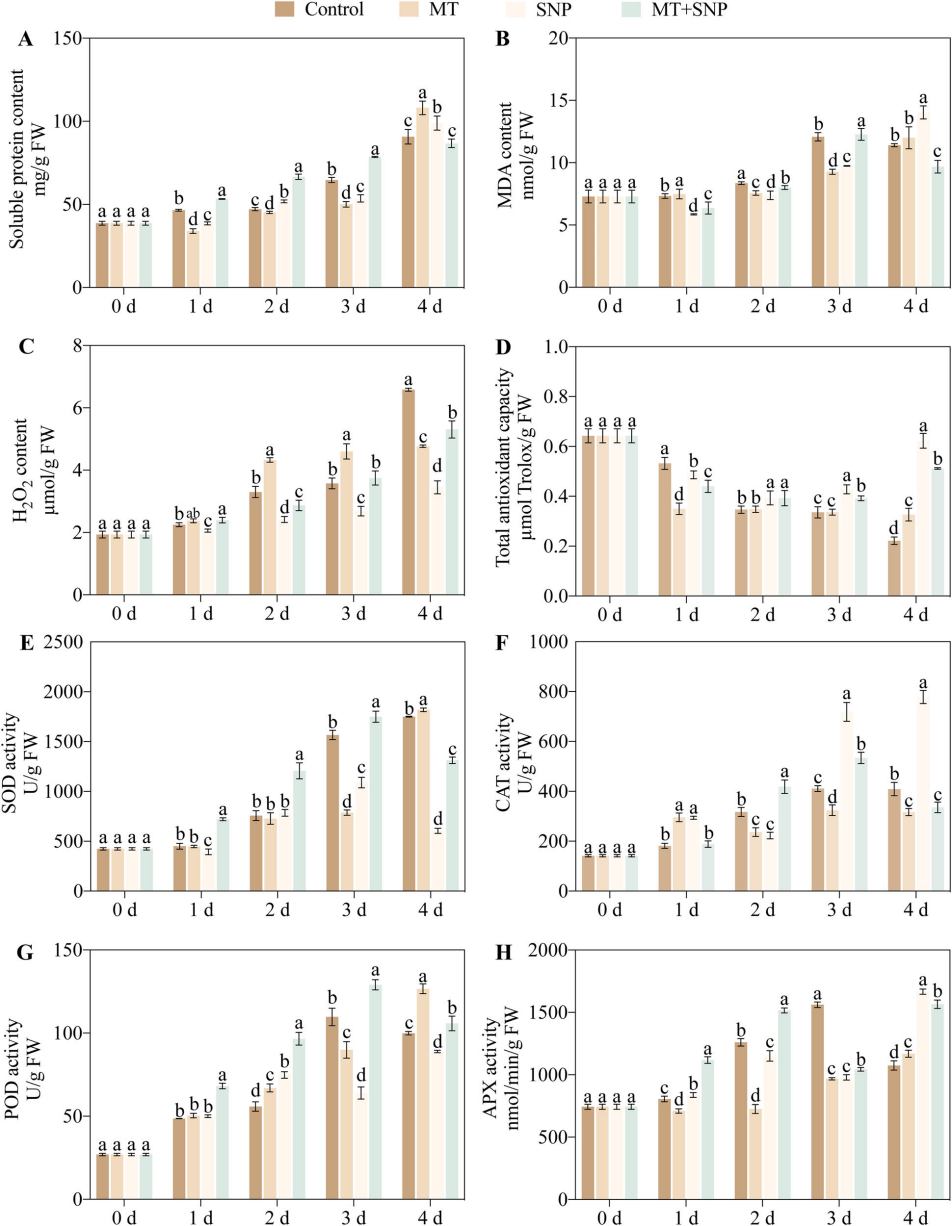
Effects of SNP and MT on antioxidant-related indicators in okra fruit after 4 d of storage. Soluble protein content (**A**), MDA content (**B**), H_2_O_2_ content (**C**), total antioxidant capacity (**D**), and the activities of SOD (**E**), CAT (**F**), POD (**G**), and APX (**H**). Error bars represent standard deviation (SD, *n* = 3). Error bars represent SD (*n* = 3). Bars with different letters within a sampling date are significantly different (*P* < 0.05).

Compared with the control, the MT treatment decreased soluble protein content by 4%–27% on 1 d–3 d and increased it by 19% on 4 d, whereas the SNP treatment reduced soluble protein content both by 17% on 1 d and 3 d, but increased it by 10% on 2 d and by 9% on 4 d (Fig. 2A). The combined MT + SNP treatment enhanced soluble protein content by 15%–41% on 1 d–3 d. For MDA content, the MT treatment increased values by 2% on 1 d and decreased them by 9% and 23% on 2 d and 3 d (Fig. 2B). In contrast, the SNP treatment lowered MDA by 12%–20% on 1 d–3 d and raised it by 23% on 4 d, while the MT + SNP treatment reduced MDA by 13%, 4%, and 15% on 1 d, 2 d, and 4 d, yet increased it by 2% on 3 d. Regarding H_2_O_2_, the MT treatment elevated its content by 31% and 29% on 2 d and 3 d and reduced it by 28% on 4 d; the SNP treatment consistently decreased H_2_O_2_ levels by 8%–48% throughout the storage period, and the MT + SNP treatment increased H_2_O_2_ by 6% on 1 d but decreased it by 13% and 19% on 2 d and 4 d (Fig. 2C). Finally, for total antioxidant capacity, the MT treatment decreased it by 34% on 1 d and increased it by 47% on 4 d; the SNP treatment decreased capacity by 9% on 1 d and increased it by 14%–181% on 2 d–4 d; and the MT + SNP treatment reduced total antioxidant capacity by 17% on 1 d and increased it by 13%–131% on 2d–4 d (Fig. 2D).

### Effects of SNP and MT on SOD, CAT, POD, and APX activities in okra fruit

Compared with the control, the MT treatment decreased SOD activity by 50% on 3 d and increased it by 2% on 4 d. The SNP treatment reduced SOD activity by 13%, 31%, and 65% on 1 d, 3 d and 4 d, whereas the combined MT + SNP treatment increased SOD activity by 12%–60% on 1d–3 d and decreased it by 24% on 4 d (Fig. 2E). For CAT activity, MT treatment increased activity by 63% on 1 d but decreased it by 21%–25% on 2 d–4 d. Conversely, the SNP treatment enhanced CAT activity by 62%, 75%, and 90% on 1 d, 3 d and 4 d, respectively, while reducing it by 30% on 2 d. The MT + SNP treatment elevated CAT activity by 32% on 2 d and 30% on 3 d, but decreased it by 18% on 4 d (Fig. 2F). Regarding POD activity, MT treatment increased POD activity by 20% on 2 d and 27% on 4 d, while reducing it by 18% on 2 d. The SNP treatment boosted POD activity by 34% on 2 d but decreased it by 42% on 3 d and 11% on 4 d. The MT + SNP treatment consistently increased POD activity by 6%–73% throughout the entire period (Fig. 2G). Concerning APX activity, MT treatment decreased activity by 12%–42% on 1 d–3 d and increased it by 9% on 4 d. The SNP treatment raised APX activity by 4% on 1 d and 55% on 4 d. In contrast, the MT + SNP treatment increased APX activity by 39%, 20%, and 46% on 1 d, 2 d and 4 d, respectively, while decreasing it by 37% on 3 d (Fig. 2H).

### Effects of SNP and MT on chlorophyll content and chlorophyll metabolism, and stability of enzyme activity in okra fruit

**Fig. 3:**
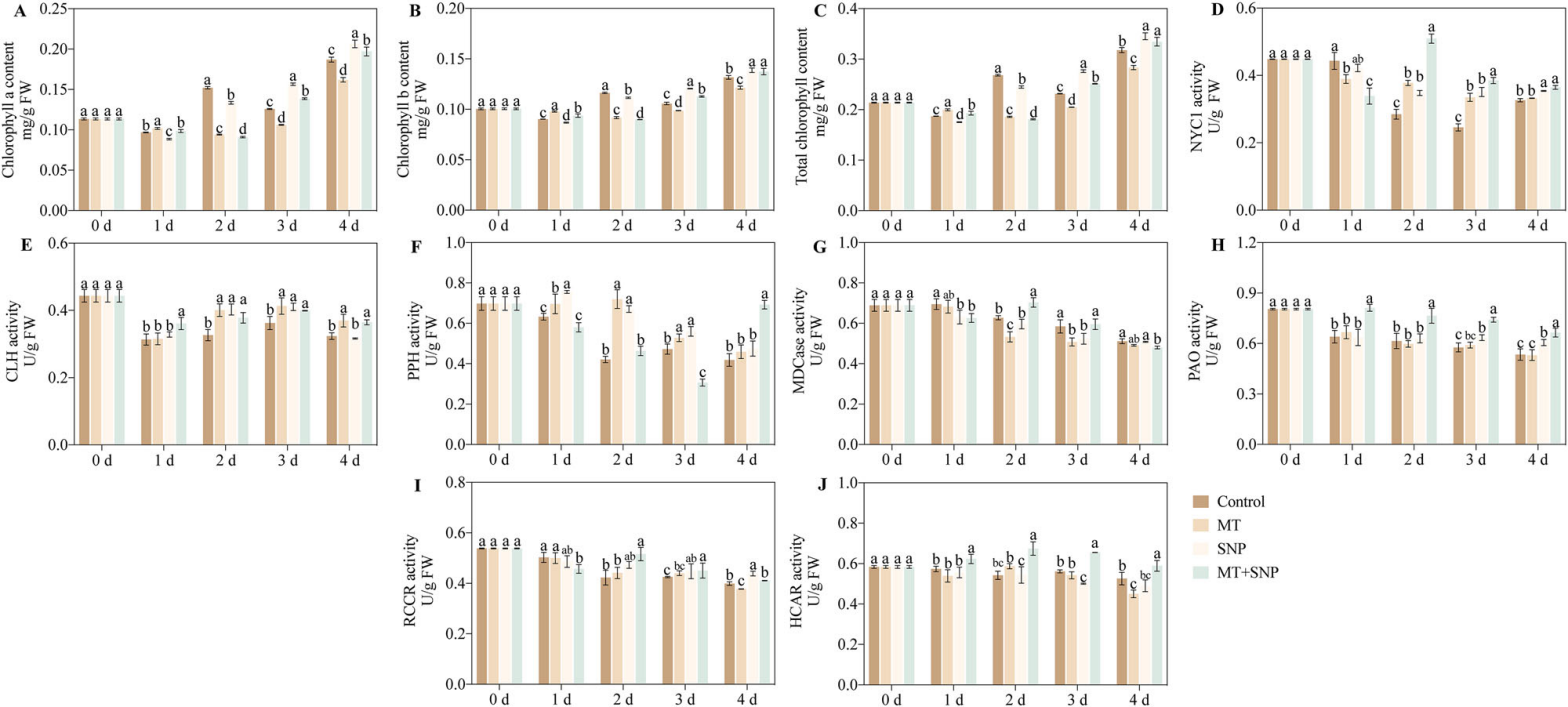
Effects of SNP and MT on chlorophyll content and chlorophyll metabolism- and stability-related enzyme activities in okra fruit after 4 d of storage. Chlorophyll a content (**A**), chlorophyll b content (**B**), total chlorophyll content (**C**), NYC1 (**D**), CLH (**E**), PPH (**F**), MDCase (**G**), PAO (**H**), RCCR (**I**), and HCAR (**J**) activities. Error bars represent SD (*n* = 3). Bars with different letters within a sampling date are significantly different (*P* < 0.05).

Compared with the control, the chlorophyll a, chlorophyll b, and total chlorophyll content of MT-treated fruit increased by 5%, 8%, and 7% on 1 d and decreased by 13%–38%, 7%–21%, and 11%–31% on 2 d–4 d, respectively (Fig. 3A–C). In SNP-treated fruit, chlorophyll a, chlorophyll b, and total chlorophyll content decreased by 9% and 12%, 4% and 4%, and 6% and 9% on 1 d and 2 d, while they increased by 24% and 10%, 14% and 5%, and 19% and 9% on 3 d and 4 d, respectively. The SNP + MT treatment increased chlorophyll a and chlorophyll b content by 10% and 7% on 3 d and by 5% and 4% on 4 d, respectively, and increased total chlorophyll content by 3%, 9%, and 5% on 1 d, 3 d and 4 d, respectively.

In okra fruit during storage, treatments with MT, SNP, and their combination (MT + SNP) produced distinct effects on enzyme activities relative to the control (Fig. 3D–J). For NYC1 activity, the MT treatment increased activity by 33% and 36% on 2 d and 3 d of storage, respectively, while both SNP and MT + SNP treatments enhanced activity by 8%–43% and 12%–79% during 2 d–4 d. For CLH activity, the MT treatment elevated activity by 14%–23% during storage 2d–4 d, and the SNP treatment increased activity by 23% and 13% on 2 d and 3 d; the MT + SNP treatment consistently enhanced CLH activity by 10%–16% throughout storage. In contrast, PPH activity was boosted by the MT and SNP treatments by 10%–71% and 19%–59%, respectively, during 1 d–3 d, with the MT + SNP treatment further increasing activity by 65% on 4 d. Regarding MDcase, the MT treatment reduced activity by 15% and 13% on storage 2 d and 3 d, and the SNP treatment decreased activity by 8% and 10% on 1 d and 3 d; the MT + SNP treatment lowered MDcase activity by 10%, 12%, and 6% on storage 1 d, 2 d and 4 d, respectively. In addition, PAO activity was increased by the SNP treatment by 10% and 13% on storage 3 d and 4 d, and by the MT + SNP treatment by 24%–29% over the entire storage period. For RRCR activity, the MT treatment decreased it by 5% on 4 d, whereas the SNP treatment increased it by 9% and 10% on 3 d and 4 d; notably, the MT + SNP treatment reduced activity by 9% on 1 d but increased it by 22% and 10% on 2 d and 3 d, respectively. Finally, HCAR activity was decreased by the MT treatment on the final storage day by 14%, and by the SNP treatment on 3 d by 11%, while the MT + SNP treatment enhanced HCAR activity by 9%–17% throughout storage.

### Transcriptome profiling and differential gene expression analysis of okra fruit under MT, SNP, and MT + SNP treatment

On the fourth day of storage, postharvest okra fruit subjected to MT, SNP, and especially the combined MT + SNP treatment exhibited a suite of improved quality indices, including lower weight loss, reduced L^*^, a^*^, and CI values with concomitant increases in b^*^, chroma, hue, and BI, enhanced soluble protein content, reduced H_2_O_2_ levels, modified MDA content, improved total antioxidant capacity, differential enzyme activities including reduced SOD, variable CAT and POD responses, elevated APX activity, and altered chlorophyll parameters—with MT reducing chlorophyll a, b, and total chlorophyll, while SNP and MT + SNP treatments increased these chlorophyll components—and modifications in chlorophyll metabolism enzymes, increase in PPH and decrease in MDcase activity. To explore the underlying molecular responses, we conducted RNA sequencing on fruit tissues from freshly harvested (FH) and both control, MT, SNP and MT + SNP-treated fruit on 4 d of storage. Fifteen cDNA libraries yielded 128.93 Gb of raw sequence data. Following removal of adapters and low-quality reads, we retained 429,092,998 high-quality reads (Q30 > 96%; Supplementary Table 2). Assembly produced 60,127 transcripts and 20,111 unigenes, with an N50 of 1928 bp and a mean length of 1743 bp (Supplementary Table 3). Unigenes were distributed as follows by length: 300–500 bp (21%), 501–1000 bp (11%), 1001–2000 bp (42%), and over 2000 bp (27%) (Supplementary Fig. 3), with 13,801 unigenes exceeding 1000 bp.

Out of the total 19,097 annotated unigenes, 5522 (29%) were between 300 and 1000 bp, while 13,575 (71%) were ≥1000 bp. Notably, the NR database provided the most comprehensive annotation with 18,954 unigenes (5444 in the 300–1000 bp range and 13,510 ≥ 1000 bp), followed by TrEMBL (18,907 unigenes; 5414 and 13,493 in the respective length categories) and eggNOG (16,540 unigenes; 4631 and 11,909). The GO database contributed annotations for 15,436 unigenes (4416 for 300–1000 bp and 11,020 for ≥1000 bp), whereas KEGG annotated 13,693 unigenes (3709 and 9984, respectively). In addition, the COG, KOG, Pfam, and SwissProt databases annotated 6167 (1316 and 4851), 11,563 (3253 and 8310), 14,877 (3367 and 11,510), and 14,541 (4095 and 10,446) unigenes, respectively (Supplementary Table 4).

Using FH samples as a baseline, pairwise comparisons were performed to identify DEGs between control and MT-, SNP-, and MT + SNP-treated fruit at four days of storage (Supplementary Table 5 and Supplementary Fig. 4). A total of 6225 DEGs (3288 upregulated and 2937 downregulated) were identified in the Control vs. FH comparison; 2426 DEGs (1187 upregulated and 1239 downregulated) in the SNP vs. Control comparison; 527 DEGs (262 upregulated and 265 downregulated) in the MT vs. Control comparison; and 256 DEGs (125 upregulated and 131 downregulated) in the MT + SNP vs. Control comparison. In addition, 4365 postharvest senescence-related DEGs were detected, of which 2175 were upregulated and 2190 were downregulated; 754 SNP-specific DEGs were identified (313 upregulated and 451 downregulated); 140 MT-specific DEGs were found (65 upregulated and 75 downregulated); and finally, 44 MT + SNP-specific DEGs were identified (13 upregulated and 31 downregulated).

To validate the transcriptomic profiling, the expression of 16 DEGs associated with four specific comparison pathways was analyzed via qRT-PCR (Supplementary Fig. 5). The gene expression patterns observed in the qRT-PCR experiments for FH, Control, MT, SNP, and MT + SNP fruit (collected on 4 d) corresponded closely with the RNA-seq data, confirming the high reproducibility and reliability of the transcriptome analysis.

### Functional analysis of four comparison-specific DEGs: GO and KEGG enrichment

**Fig. 4:**
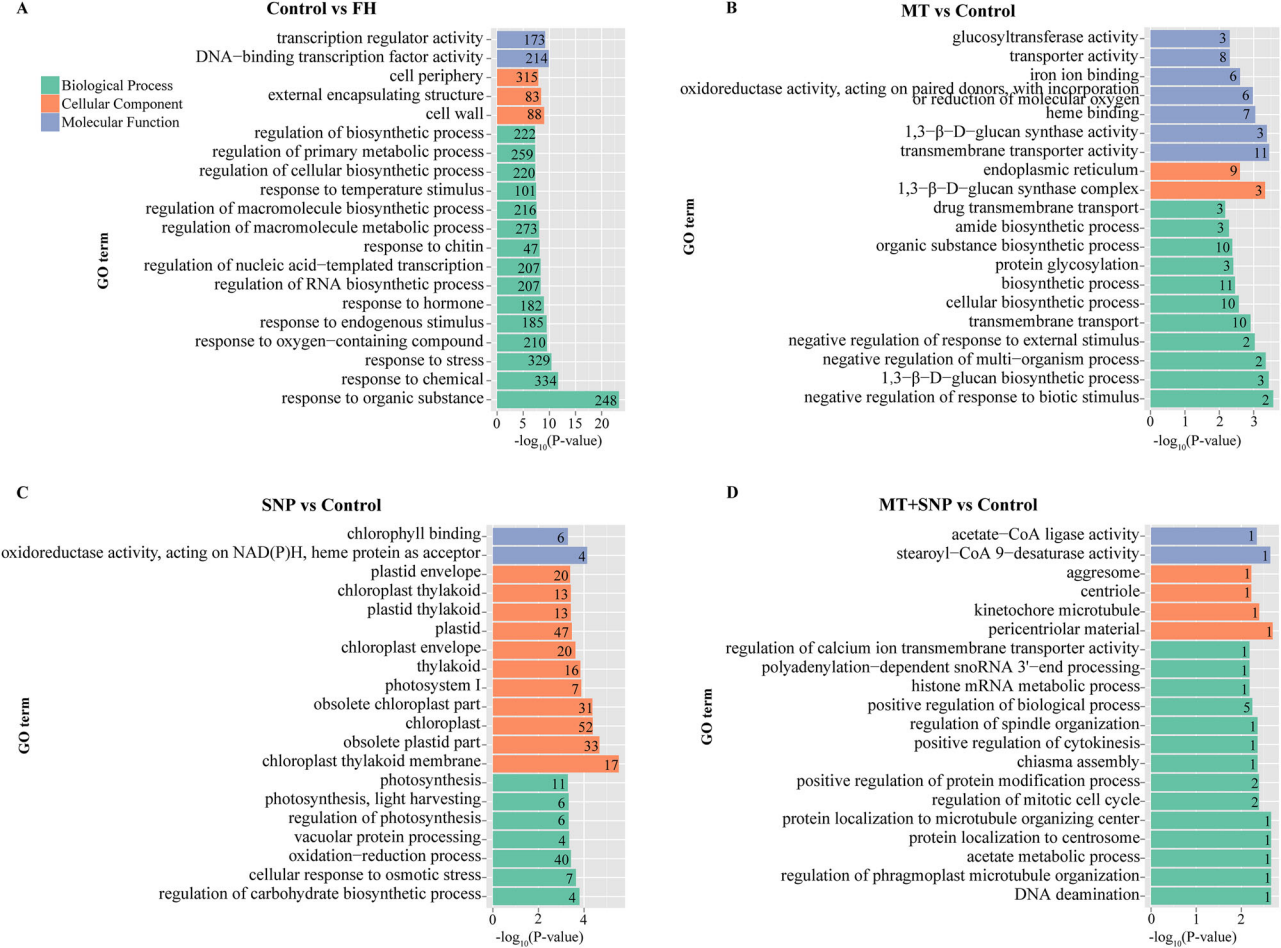
GO terms significantly enriched in the specific DEGs of okra fruit in four comparison groups. Control vs. FH (**A**), MT vs. Control (**B**), SNP vs. Control (**C**), and MT + SNP vs. Control (**D**).

We conducted a Gene Ontology (GO) enrichment analysis to elucidate the functions of specific DEGs in okra fruit on 4 d of storage (Fig. 4 and Supplementary Data 1). The predominant GO categories identified were “response to organic substance”, “cell wall”, and “DNA-binding transcription factor activity” in the Control vs. FH group (Fig. 4); “1,3-β-D-glucan biosynthetic process”, “organic substance biosynthetic process”, and “oxidoreductase activity” in the MT vs. Control group; “oxidation−reduction process”, “chloroplast thylakoid membrane”, and “oxidoreductase activity” in the SNP vs. Control group; and “positive regulation of biological process” in the SNP + MT vs. Control group, all of which are indicative of roles in antioxidant defense and chlorophyll metabolism.

**Fig. 5:**
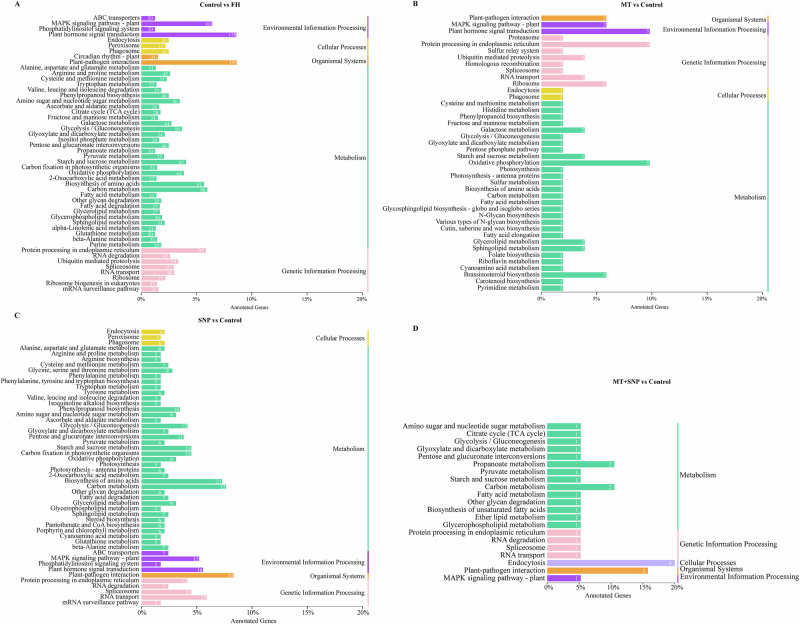
KEGG analysis of specific DEGs of okra fruit in four comparison groups. Control vs. FH (**A**), MT vs. Control (**B**), SNP vs. Control (**C**), and MT + SNP vs. Control (**D**).

In addition, the DEGs specific to each of the four comparisons were mapped to pathways in the KEGG database. In the Control vs. FH group, the most enriched pathways were plant-pathogen interaction, plant hormone signal transduction, and MAPK signaling pathway–plant (Fig. 5 and Supplementary Data 2). In the MT vs. Control group, oxidative phosphorylation, protein processing in the endoplasmic reticulum, and plant hormone signal transduction were most enriched. For the SNP vs. Control group, the top pathways were plant-pathogen interaction, carbon metabolism, and biosynthesis of amino acids. Finally, in the MT+SNP vs. Control group, endocytosis, plant-pathogen interaction, and propanoate metabolism were the most enriched pathways.

### Differential expression of antioxidant defense-related DEGs

Although antioxidant pathways did not rank among the top ten KEGG-enriched pathways for DEGs specific to the four comparison groups, thirty-six DEGs associated with redox homeostasis were selected to investigate the molecular mechanisms underlying postharvest senescence and reactive oxygen species (ROS) mitigation elicited by exogenous MT and SNP. On 4 d of storage, okra fruit exhibited upregulation of eight *PODs*, one *APX1*, three monodehydroascorbate reductases (*MDHARs*), and five glutathione S-transferases (*GSTs*), whereas two *CAT2*, nine *PODs*, one MDHAR, four GSTs, and two ferredoxins (*Frxs*) were downregulated. Under MT treatment alone, only one *MDHAR* was downregulated in postharvest okra fruit. Under SNP treatment, two *PODs*, three *MDHARs*, two *GSTs*, and one *Frx2* were upregulated, while eight *PODs* and one *GSTF9* were downregulated. Similarly, under combined MT and SNP treatment, one *POD42* was upregulated and one *MDHAR* was downregulated (Table 1).

**Table 1:** List of selected genes that may be responsible for SNP- and MT-mediated postharvest quality of okra fruit

Gene id	Gene name	Log2 Fold-Change				Gene description
		Control vs. FH	MT vs. Control	SNP vs. Control	MT + SNP vs. Control	
Antioxidant defense-related DEGs						
TRINITY_DN5801_c0_g3	*CAT2*	−0.74	-	-	-	Catalase isozyme 2
TRINITY_DN7862_c0_g1	*CAT2*	−0.67	-	-	-	Catalase isozyme 2
TRINITY_DN9274_c0_g1	*POD5*	−2.55	-	-	-	Peroxidase 5
TRINITY_DN8559_c0_g1	*POD11*	11.26	-	−2.80	-	Peroxidase 11
TRINITY_DN3291_c0_g1	*POD17*	−1.72	-	1.37	-	Peroxidase 17
TRINITY_DN8861_c0_g1	*POD21*	−2.87	-	2.97	-	Peroxidase 21
TRINITY_DN15109_c0_g1	*POD25*	2.66	-	−1.73	-	Peroxidase 25
TRINITY_DN71851_c0_g1	*POD25*	3.19	-	-	-	peroxidase 25
TRINITY_DN5145_c0_g1	*POD31*	1.89	-	−1.55	-	peroxidase 31-like
TRINITY_DN34030_c0_g1	*POD40*	3.96	-	-	-	peroxidase 40 precursor
TRINITY_DN22974_c0_g1	*POD42*	−1.13	-	−1.53	0.72	Peroxidase 42
TRINITY_DN26603_c0_g1	*POD54*	−2.26	-	-	-	Peroxidase 54
TRINITY_DN216153_c0_g1	*POD54*	4.31	-	−4.58	-	Peroxidase 54
TRINITY_DN105024_c0_g1	*POD55*	2.29	-	−2.83	-	Peroxidase 55
TRINITY_DN8076_c0_g1	*POD64*	−3.71	-	-	-	PREDICTED: peroxidase 64-like
TRINITY_DN9372_c0_g1	*POD64*	−1.57	-	-	-	Peroxidase 64-like
TRINITY_DN1208_c1_g3	*POD66*	−5.16	-	-	-	peroxidase 66
TRINITY_DN10147_c1_g1	*POD73*	−2.71	-	-	-	Peroxidase 73
TRINITY_DN14032_c0_g1	*PODP7*	1.31	-	−2.07	-	PREDICTED: peroxidase P7-like isoform X2
TRINITY_DN7047_c0_g1	*PODP7*	-	-	−2.00	-	PREDICTED: peroxidase P7-like
TRINITY_DN3376_c0_g1	*APX1*	0.68	-	-	-	L-ascorbate peroxidase 1, cytosolic
TRINITY_DN147219_c0_g1	*MDHAR*	−1.42	-	2.27	−0.76	Monodehydroascorbate reductase
TRINITY_DN1764_c0_g1	*MDHAR*	−0.86	-	-	-	Monodehydroascorbate reductase
TRINITY_DN465_c0_g1	*MDHAR*	0.10	−0.59	0.96	-	Monodehydroascorbate reductase
TRINITY_DN2663_c0_g1	*MDHAR3*	−1.38	-	1.35	-	Bifunctional monodehydroascorbate reductase and carbonic anhydrase nectarin-3-like
TRINITY_DN25863_c0_g2	*GST*	−2.54	-	1.59	-	Glutathione s-transferase-like protein
TRINITY_DN40077_c0_g1	*GST*	4.10	-	-	-	Glutathione S-transferase family protein isoform 1
TRINITY_DN6347_c0_g2	*GST*	−2.02	-	-	-	PREDICTED: glutathione S-transferase zeta class-like
TRINITY_DN297_c0_g1	*GST1*	2.13	-	-	-	Glutathione S-transferase family protein isoform 1
TRINITY_DN29445_c0_g2	*GST7*	2.17	-	-	-	Putative Glutathione S-transferase tau 7
TRINITY_DN2034_c0_g1	*GSTF9*	0.85	-	−0.78	-	Glutathione S-transferase F9-like
TRINITY_DN4089_c0_g1	*GST-DHAR4*	1.69	-	-	-	Putative glutathione S-transferase DHAR4
TRINITY_DN6588_c1_g1	*GST-PARB*	−1.74	-	-	-	Glutathione S-transferase PARB
TRINITY_DN86442_c0_g1	*GSTU7*	−2.00	-	3.13	-	Glutathione S-transferase U7
TRINITY_DN5794_c0_g1	*Frx2*	−2.08	-	1.41	-	Ferredoxin-2
TRINITY_DN18174_c0_g1	*Frx3*	−1.80	-	-	-	Ferredoxin-3
Chlorophyll metabolism-related DEGs						
TRINITY_DN32450_c0_g1	*Lhcb*	−3.01	-	2.78	-	Chlorophyll a-b binding protein, chloroplastic-like
TRINITY_DN13078_c0_g1	*Lhcb3*	−2.12	-	2.15	-	Chlorophyll a-b binding protein 3
TRINITY_DN34119_c0_g1	*Lhcb4.2*	-	-	2.19	-	Chlorophyll a-b binding protein CP29.2
TRINITY_DN16265_c0_g1	*Lhcb5*	−1.56	-	2.45	-	PREDICTED: chlorophyll a-b binding protein CP26, chloroplastic
TRINITY_DN16878_c1_g1	*Lhcb5*	-	-	3.94	-	PREDICTED: chlorophyll a-b binding protein of LHCII type 1-like
TRINITY_DN48304_c0_g1	*Lhcb5*	-	−1.65	2.02	-	chlorophyll a-b binding protein of LHCII type 1-like
TRINITY_DN12532_c0_g1	*Lhcb5*	-	-	2.60	-	PREDICTED: chlorophyll a-b binding protein of LHCII type 1-like
TRINITY_DN1071_c0_g1	*Lhcb6*	-	-	1.74	-	Chlorophyll a-b binding protein 6, chloroplastic
TRINITY_DN14406_c0_g1	*Lhcb6*	-	-	2.36	-	PREDICTED: chlorophyll a-b binding protein 6, chloroplastic
TRINITY_DN54967_c0_g1	*Lhcb6*	−2.68	-	2.33	-	Chlorophyll a-b binding protein
TRINITY_DN127_c0_g1	*Lhcb7*	−1.90	−1	2.02	-	PREDICTED: chlorophyll a-b binding protein 7, chloroplastic
TRINITY_DN10806_c0_g1	*Lhcb151*	-	-	1.76	-	PREDICTED: chlorophyll a-b binding protein 151, chloroplastic
TRINITY_DN9711_c1_g1	*ChlB*	1.28	-	-	-	Light-independent protochlorophyllide reductase subunit B
TRINITY_DN9711_c1_g1	*ChlB*	1.28	-	-	-	Light-independent protochlorophyllide reductase subunit B
TRINITY_DN11720_c0_g2	*SGR*	8.32	-	-	-	Protein STAY-GREEN
TRINITY_DN1344_c0_g1	*HO1*	3.12	-	-	-	Heme oxygenase 1
TRINITY_DN1484_c0_g1	*HO1*	−1.26	-	-	-	Heme oxygenase-like, multi-helical isoform 1
TRINITY_DN90537_c0_g1	*HY2*	−1.27	-	-	-	Phytochromobilin:ferredoxin oxidoreductase
TRINITY_DN5896_c0_g1	*GGDR*	−1.92	-	-	-	Geranylgeranyl diphosphate reductase
TRINITY_DN11844_c0_g1	*POR*	-	-	2.30	-	Protochlorophyllide reductase
TRINITY_DN13487_c0_g1	*POR*	−2.27	-	-	-	Protochlorophyllide reductase
TRINITY_DN10593_c0_g1	*POR-like*	−2.76	-	2.13	-	PREDICTED: protochlorophyllide reductase-like
TRINITY_DN4391_c0_g1	*PAO*	-	-	−0.97	-	Pheophorbide a oxygenase
TRINITY_DN8481_c0_g1	*HCAR*	-	-	1.56	-	7-hydroxymethyl chlorophyll a reductase
Transcription factors and protein kinases						
TRINITY_DN1096_c0_g1	*RAP2-4*	−0.73	-	-	-	Ethylene-responsive transcription factor RAP2-4
TRINITY_DN11501_c1_g1	*DREB1A*	4.69	-	-	-	Dehydration-responsive element-binding protein 1 A
TRINITY_DN6395_c0_g1	*ERF3*	-	-	0.98	-	Ethylene-responsive transcription factor 3
TRINITY_DN8242_c0_g1	*ERF4*	1.27	-	-	-	Ethylene-responsive transcription factor 4
TRINITY_DN3772_c0_g1	*ERF4*	2.13	-	-	-	PREDICTED: ethylene-responsive transcription factor 4-like
TRINITY_DN3772_c0_g2	*ERF4*	1.18	-	-	-	PREDICTED: ethylene-responsive transcription factor 4-like
TRINITY_DN8058_c0_g1	*ERF5*	-	-	2.13	-	Ethylene-responsive transcription factor 5
TRINITY_DN19693_c0_g1	*ERF9*	2.74	-	-	-	PREDICTED: ethylene-responsive transcription factor 9
TRINITY_DN12966_c0_g1	*ERF011*	1.13	-	-	-	ERF011 protein
TRINITY_DN3805_c0_g1	*ERF017*	11.41	-	-	-	ERF017 protein
TRINITY_DN8459_c0_g1	*ERF017*	2.77	-	-	-	ERF017 protein
TRINITY_DN9173_c0_g1	*ERF025*	3.29	-	-	-	ERF025 protein
TRINITY_DN114837_c0_g1	*ERF27*	-	-	−3.88	-	ERF027 protein
TRINITY_DN214995_c0_g1	*ERF061*	1.15	-	-	-	ethylene-responsive transcription factor ERF061-like
TRINITY_DN152802_c0_g1	*ERF112*	8.38	-	-	-	ERF112 protein
TRINITY_DN6479_c0_g1	*ERF112*	1.96	-	-	-	ERF112 protein
TRINITY_DN2798_c0_g1	*ERF113*	1.12	-	-	-	ERF113 protein
TRINITY_DN8359_c0_g1	*ERF1A*	3.78	-	-	-	Ethylene-responsive transcription factor 1A
TRINITY_DN6812_c0_g1	*CRF1*	−4.76	-	-	-	PREDICTED: ethylene-responsive transcription factor CRF1
TRINITY_DN66856_c0_g1	*CRF2*	1.50	-	-	-	Ethylene-responsive transcription factor CRF2
TRINITY_DN6812_c0_g2	*CRF3*	3.01	-	-	-	Ethylene-responsive transcription factor CRF3
TRINITY_DN8882_c0_g1	*TOE3*	−1.47	-	-	-	AP2-like ethylene-responsive transcription factor TOE3
TRINITY_DN9903_c0_g1	*DREB1*	−1.21	-	-	-	PREDICTED: ethylene-responsive transcription factor RAP2-1-like
TRINITY_DN9237_c0_g1	*DREB2E*	1.90	-	-	-	Dehydration-responsive element-binding protein 2E
TRINITY_DN5597_c0_g1	*WRKY4*	-	−1.54	-	-	PREDICTED: LOW QUALITY PROTEIN: probable WRKY transcription factor 4
TRINITY_DN8258_c0_g1	*RAX3*	-	−1.20	-	-	Transcription factor RAX3
TRINITY_DN13605_c0_g1	*RAP2-3*	-	-	1.18	-	PREDICTED: ethylene-responsive transcription factor RAP2-3
TRINITY_DN6318_c0_g1	*RAP2-10*	-	-	0.93	-	ethylene-responsive transcription factor RAP2-10-like
TRINITY_DN25001_c0_g1	*bHLH106*	-	-	-	3.25	bHLH106 protein
TRINITY_DN3188_c0_g1	*NAC90*	-	-	-	−1.88	PREDICTED: NAC domain-containing protein 90-like
TRINITY_DN1740_c0_g1	*SRF6*	-	-	-	0.93	Protein STRUBBELIG-RECEPTOR FAMILY 6
TRINITY_DN16448_c0_g1	*SRF7*	-	-	-	0.89	Protein STRUBBELIG-RECEPTOR FAMILY 7
TRINITY_DN21988_c0_g1	*PMEI*	-	-	-	1.76	invertase/pectin methylesterase inhibitor family protein
TRINITY_DN41399_c0_g1	*PTI1-3*	-	-	-	−1.05	PTI1-like tyrosine-protein kinase 3

### Differential expression of chlorophyll metabolism-related DEGs

We identified 24 candidate DEGs related to chlorophyll metabolism across four comparison groups. At the end of storage, one each of *ChlB*, *SGR*, and *HO1* was upregulated, whereas five chlorophyll a‑b binding proteins (*Lhcbs*), one *HO1*, one *HY2*, one *GGDR*, and two *PORs* (*POR‑like* and *POR*) were downregulated. Under MT treatment of okra fruit, *Lhcb7* and *Lhcb5* were downregulated. Under SNP treatment, 12 *Lhcbs*, two *PORs*, and one *HCAR* were upregulated, while one *PAO* was downregulated.

### Differential expression of transcription factors and protein kinases

We identified 1867 DEGs associated with transcription factors and protein kinases (Supplementary Fig. 6). Specifically, 483 DEGs were found in the Control vs. FH group, 11 in the MT vs. Control group, 69 in the SNP vs. Control group, and 6 in the MT + SNP vs. Control group (Supplementary Data 3). Of these, 237 were transcription factors (TFs), 51 were transcription regulators (TRs), and 195 were protein kinases (PKs). In the Control vs. FH group, the majority of differentially expressed genes belonged to the AP2/ERF-ERF gene family, with 17 genes upregulated and four downregulated (Table 1). Moreover, *WRKY4* and *RAX3*, members of the MYB family, were downregulated by MT treatment. Similarly, in the Control vs FH group, an additional analysis of the AP2/ERF-ERF gene family revealed five upregulated genes and one downregulated gene (*ERF27*). Finally, four PKs and two TFs were differentially expressed; specifically, *bHLH106*, *SRF6*, *SRF7*, and *PMEI* were upregulated, while *NAC90* and *PTI1-3* were downregulated.

### Gene module analysis in MT and SNP-treated okra fruit

**Fig. 6:**
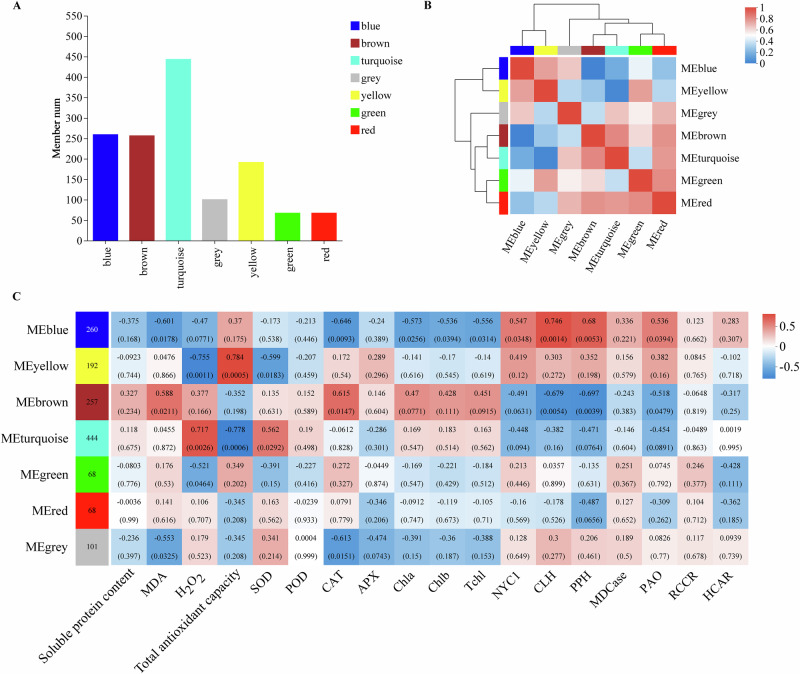
WGCNA and identification of antioxidant defense- and chlorophyll metabolism-related genes, transcription factors, and protein kinases. **A** The number of co-expressed genes in different modules (by color). **B** Hierarchical clustering dendrogram (upper panel) and correlation heatmap (lower panel) of module eigengenes (ME) to examine higher-order relationships between the modules. **C** Heatmap of correlations with antioxidant defense- and chlorophyll metabolism-related physiological indicators.

Using WGCNA, we examined how genes induced by MT and SNP are regulated during okra fruit postharvest senescence. Our study focused on genes involved in antioxidant defense and chlorophyll metabolism (Table 1), as well as their TFs and PKs (Supplementary Data 3). We identified seven distinct co‑expression modules (Fig. 6A), which were organized into two meta‑modules based on their correlation patterns (Fig. 6B). Meta1 comprised the blue and yellow modules, while Meta2 included the gray, brown, turquoise, green, and red modules. Within each meta‑module, constituent modules exhibited positive correlations; however, Meta1 showed negative associations with H_2_O_2_, SOD, chlorophyll a, chlorophyll b, and total chlorophyll, while correlating positively with NYC1, CLH, PPH, MDCase, PAO, and HCCR activities. Moreover, the gray, brown, turquoise, green, and red modules demonstrated both negative and positive relationships with antioxidant defense and chlorophyll metabolism (Fig. 6C). These modules exhibited strong positive and negative correlations with total antioxidant capacity and CLH activity.

**Fig. 7:**
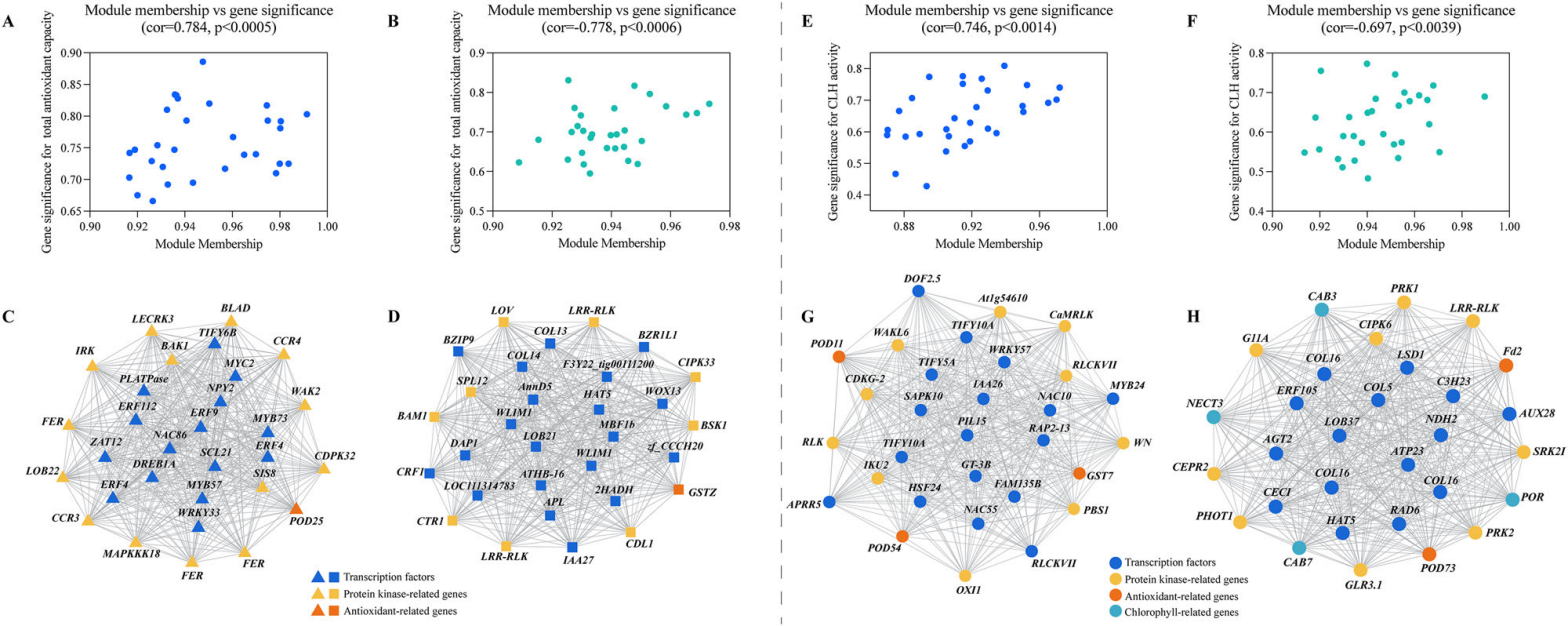
Scatterplots of module membership vs. gene significance and Cytoscape representation of the module of genes of interest (MGI). (**A**) Yellow; (**B**) Turquoise; (**E**) Blue; (**F**) Brown. Cytoscape representations of MGI within the yellow (**C**), turquoise (**D**), blue (**G**), and brown (**H**) modules. Edges with a weight above 0.02 are shown.

To further explore the relationships among total antioxidant capacity, CLH, and these modules, we filtered transcripts from the yellow, turquoise, blue, and brown modules that simultaneously displayed the highest gene significance (GS) and module membership (MM) (Fig. 7A, B, E, F). Figure 7B, C, G, H illustrates the interactions between transcription factors and genes involved in antioxidant defense and chlorophyll metabolism within those four modules (Supplementary Data 4). In the yellow module, the three highest‑degree genes were *NAC86*, *FER*, and *ERF4*; these were co‑expressed with *POD25* (Supplementary Data 5). Conversely, in the turquoise module, the top three highest‑degree genes were *zf_CCCH20*, *HAT5*, and *APL*, which were co‑expressed with *GSTZ*. In the blue module, the three highest‑degree genes were *MYB24*, *GT-3B*, and *FAM135B*; these were co‑expressed with *POD11*, *POD25*, and *GST7*. In the brown module, the top three highest‑degree genes were *COL16*, *AUX28*, and *CEPR2*; these were co‑expressed with *POD73*, *NECT3*, *Fd2*, *CAB3*, *CAB7*, and *POR*. These results suggest that MT and SNP modulate okra fruit postharvest senescence by promoting the co‑expression of transcription factors and genes associated with antioxidant defense and chlorophyll metabolism—a relationship that warrants further investigation.

## Discussion

Postharvest okra fruit, like many other horticultural commodities, is prone to rapid quality deterioration characterized by weight loss, color changes, and loss of nutritional and antioxidant properties^1^. In the present study, SNP and MT treatments, alone or in combination, effectively suppressed weight loss, mitigated color degradation, and enhanced antioxidant capacity during storage. Notably, the combined MT + SNP treatment exerted the most pronounced effects, underscoring the potential of these two signaling molecules to synergistically preserve the chlorophyll stability and nutritional quality of okra fruit. Weight loss in postharvest fruit is predominantly driven by water evaporation and metabolic processes that alter cell wall polysaccharides and membrane phospholipids^1,3^. Consistent with earlier findings in mango^4^ and papaya^5^, exogenous application of MT and NO effectively reduced weight loss in okra fruit, with the combined application of MT + SNP showing an even stronger effect. This observation suggests that simultaneous enhancement of antioxidative pathways and maintenance of membrane lipid integrity could help preserve cellular compartmentalization and reduce water loss during storage^24^. The mechanisms likely involve protection of membrane phospholipids against peroxidation and preservation of cell wall polysaccharide integrity, which collectively maintain tissue structure and water retention capacity. The visual appearance of okra fruit, particularly its color parameters, significantly influences consumer acceptance and market value. In the present study, all treatments (MT, SNP, and especially MT + SNP) contributed to lower L^*^, a^*^, and CI values and higher b^*^, chroma, hue, and BI values, preserving desirable color attributes compared with controls. These color metrics directly reflect the stability of chlorophyll molecules and related pigments, which are susceptible to oxidative degradation during postharvest storage. Similar effects on maintaining color-related phytochemicals have been reported in other perishable produce, such as mango treated with phytohormones or antioxidants^4,25^. The improved color retention in MT + SNP-treated okra fruit likely results from reduced chlorophyll degradation and oxidative stress, reflecting protective mechanisms that maintain pigment molecular stability and preserve the visual quality attributes valued by consumers^26,27^.

Postharvest senescence is biochemically characterized by the overproduction of ROS, which leads to lipid peroxidation of membrane phospholipids and subsequent cellular damage^7^. Our results indicated that NO and MT treatments modulated oxidative stress indicators, specifically MDA and H_2_O_2_ contents, while enhancing total antioxidant capacity. Particularly on 4 d, combined MT + SNP treatment maintained comparatively lower levels of H_2_O_2_ and MDA, highlighting its stronger ability to scavenge ROS and mitigate lipid peroxidation of membrane components. The chemical basis for this protection likely involves both direct scavenging of free radicals and the modulation of enzymatic antioxidant systems. Similar protective effects on cellular redox homeostasis have been noted when exogenous salicylic acid, auxin, glutathione, and ascorbic acid were used to strengthen the enzymatic antioxidant system in winter jujube, mango, tomato, and papaya^4,5,28^. Enzymatic antioxidants (SOD, CAT, POD, and APX) constitute a critical line of defense against ROS by catalyzing specific redox reactions that neutralize potentially harmful oxidative species^29^. In okra fruit, SNP and MT treatments differentially influenced the activities of these enzymes through complex regulatory mechanisms. Although some individual treatments led to transient declines in specific enzymes (e.g., reduced SOD and CAT activity in certain storage stages), the combined MT + SNP treatment generally enhanced POD and APX activities while maintaining balanced SOD and CAT levels. Such cooperative regulation of multiple enzymatic systems might explain the superior ROS scavenging capacity observed in MT + SNP-treated fruit. Previous work in mango suggested that co-expression of TFs, such as *bZIP* and *ERFs*, with antioxidant enzyme genes contributed to the regulation of ROS homeostasis^4,30^. Similarly, in okra, our transcriptome data revealed that genes encoding *PODs*, *GSTs*, and *MDHARs* were differentially expressed in response to MT and SNP, thereby reinforcing enzymatic and non-enzymatic antioxidative networks that protect valuable bioactive compounds from oxidative degradation^31^.

Chlorophyll degradation is a hallmark of postharvest senescence in green vegetables, leading to color changes that often reduce market value and nutritional quality^9^. At the molecular level, this process involves a cascade of enzymatic reactions that transform chlorophyll into colorless catabolites. In this study, exogenous MT and SNP delayed chlorophyll breakdown by modulating key enzymes involved in chlorophyll catabolism, such as PPH, CLH, and PAO. While MT alone sometimes showed reduced chlorophyll contents on certain days, SNP consistently increased chlorophyll a, chlorophyll b, and total chlorophyll, particularly at later storage stages. These findings align with reports that exogenous MT and SNP can retard chlorophyll degradation in cabbage and other horticultural produce by interfering with the activity of chlorophyll-degrading enzymes^23,32^. Furthermore, the increase in CLH, PPH, PAO, and related genes under combined treatment suggests a complex regulatory mechanism that accelerates pigment turnover while simultaneously conserving overall chlorophyll content to retain greener coloration^33^. Interestingly, reduced MDcase activity under MT + SNP treatment may also contribute to stabilizing chlorophyll, as MDcase (mesophyll-derived cell death-related enzyme) has been associated with tissue senescence and chlorophyll degradation^34^. These data collectively imply that molecular crosstalk between NO and MT fine-tunes chlorophyll metabolism, slows down color loss, and maintains both the visual appeal and nutritional quality of okra fruit^9^. The preservation of chlorophyll is particularly important from a Food Chem.perspective, as these pigments not only contribute to color but also possess antioxidant properties and are associated with other bioactive compounds that enhance the nutritional value of okra.

RNA-seq analysis confirmed the biochemical trends by identifying DEGs associated with redox homeostasis, chlorophyll metabolism, and transcriptional regulation at the molecular level. Under combined MT + SNP treatment, the number of uniquely regulated DEGs (44 in total) was lower than that observed for either treatment alone, suggesting that NO and MT share overlapping downstream targets while also generating synergistic effects on gene expression networks^35,36^. Notable changes included the upregulation of POD- and GST-encoding genes, consistent with increased enzymatic activities that promote ROS scavenging and detoxification of oxidation products^4^. In contrast, genes encoding certain *Frxs* and *CATs* were downregulated, supporting the observed transient decreases in CAT activity at specific time points. These differential expression patterns reflect the complex biochemical coordination required to maintain cellular redox balance during extended storage. With respect to chlorophyll metabolism, the present transcriptome data highlighted the differential expression of *Lhcbs*, *POR*, *PAO*, and *HCAR*, underscoring their pivotal roles in regulating chlorophyll biosynthesis and degradation pathways^14,22^. The upregulation of *POR-like* in SNP-treated fruit suggests an enhancement of chlorophyll synthesis pathways, whereas the downregulation of a *PAO* in the same group indicates attenuated chlorophyll breakdown. Meanwhile, the MT + SNP treatment led to selective overexpression of *PAO*, implying a more dynamic modulation of pigment turnover. These seemingly contradictory patterns point to a coordinated molecular mechanism allowing precise control of chlorophyll homeostasis through balanced regulation of both biosynthetic and catabolic pathways^14,22,37^.

TFs such as *AP2*/*ERF*, *MYB*, and *NAC* families often function as global regulators of postharvest physiological processes and biochemical pathways^18^. Our weighted gene co-expression network analysis revealed that key TFs—such as *NAC86*, *ERF4*, *MYB24*, and *GT-3B*—were co-expressed with antioxidant- and chlorophyll-related genes, suggesting their role as master regulators of multiple quality-related pathways. Similar findings in other fruit systems have shown that TFs directly or indirectly modulate the expression of antioxidative enzymes, cell wall-modifying enzymes, and senescence-associated genes that collectively determine postharvest quality retention^38,39^. Here, co-expression in the blue, yellow, turquoise, and brown modules suggests that NO and MT may converge on shared regulatory nodes, enhancing the transcription of genes that bolster antioxidant capacity and chlorophyll retention (Quesada et al., 2009; Upadhyay et al., 2023). Moreover, protein kinases, integral to numerous signal transduction cascades, were differentially expressed under MT and SNP treatments, further implying that multi-level regulation underpins the synergistic effects observed^33,40^. These protein kinases likely facilitate the phosphorylation-dependent activation of transcription factors and metabolic enzymes, thereby connecting external chemical signals (SNP and MT) to specific biochemical responses that preserve food quality attributes. The identification of these regulatory hubs provides potential targets for future postharvest interventions aimed at enhancing the chlorophyll stability, nutritional value, and shelf life of okra and similar perishable produce.

**Fig. 8:**
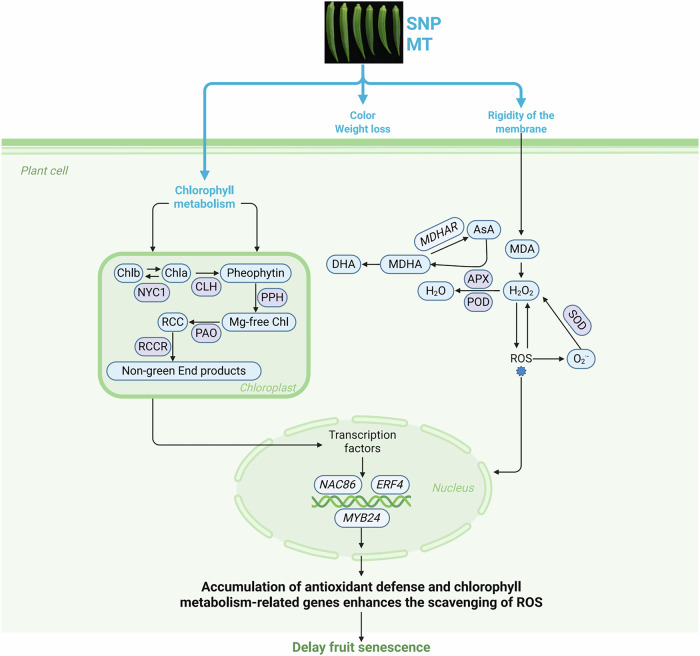
A model of okra fruit postharvest senescence as mediated by SNP and MT.

This study demonstrates that MT and SNP treatments effectively preserve the chlorophyll stability and quality attributes of postharvest okra fruit (Fig. 8). These treatments reduced the weight loss while maintaining desirable color parameters, including lower L^*^ and a^*^ values and higher b^*^, chroma, hue, and BI values, which directly reflect the preservation of pigment molecules and tissue integrity. Biochemical analysis revealed that MT and SNP enhanced the antioxidant defense system by modulating SOD, CAT, POD, and APX enzyme activities, thereby reducing MDA and H_2_O_2_. Notably, the combined MT + SNP treatment exhibited synergistic effects in preserving chlorophyll a, chlorophyll b, and total chlorophyll content through selective regulation of key enzymes in chlorophyll metabolism (particularly PPH and MDcase). Transcriptome analysis further elucidated the molecular mechanisms underlying these biochemical changes, revealing complex gene expression networks involving antioxidant-related genes (*PODs*, *GSTs*, *MDHARs*), chlorophyll metabolism genes (*POR*, *PAO*, *Lhcbs*), and their upstream regulators (*NAC86, ERF4*, *MYB24*). The co-expression patterns identified through WGCNA highlighted the integrated nature of redox homeostasis and pigment metabolism pathways in maintaining postharvest quality. Our findings provide evidence for the molecular mechanisms by which SNP and MT treatments preserve specific phytochemicals, including chlorophyll pigments, antioxidant molecules, and the visual quality of okra fruit, suggesting potential molecular pathways for developing postharvest technologies aimed at extending shelf life while maintaining the functional food properties of fresh produce. However, several limitations should be acknowledged. First, this study was conducted under controlled laboratory conditions (20 °C, 80–90% humidity), and the efficacy of these treatments under varying commercial storage conditions remains to be validated. Second, the economic feasibility and scalability of MT and SNP applications in commercial postharvest operations require further investigation. Third, our transcriptomic analysis was limited to a single time point (day 4), which may not capture the complete temporal dynamics of gene expression changes throughout storage. Fourth, while we identified key regulatory pathways, the functional validation of specific genes and their roles in preservation mechanisms warrants additional research.

## Methods

### Plant materials and treatments

‘Lvba’ okra (*Abelmoschus esculentus* L.) fruit at mature stages, characterized by full physiological maturity, a color change, and the onset of seed development, were harvested from an experimental station in Zhanjiang, China (Fig. 1A). Uniform, defect-free fruit were sanitized with 0.01% sodium hypochlorite for 5 min, air-dried, and immersed for 5 min in one of four solutions: (1) distilled water (Control), (2) 100 µM melatonin (MT), (3) 0.5 mM sodium nitroprusside (SNP), or (4) MT + SNP at these same concentrations. 0.5 mM SNP and 100 µM MT were identified as the most effective concentrations for preserving okra fruit quality, minimizing weight loss and maintaining fruit peel color throughout storage (Supplementary Figs. 1 and 2). Tween-80 (1:1000, v/v) was added to each solution. After treatment, fruit were air-dried at ambient temperature, then stored at 20 °C and 80-90% relative humidity (RH) for up to four days. Every treatment had 180 fruits, which were used fresh, and three biological replicate samples for every indicator analysis were used. Each biological replicate consisted of 60 fruit randomly selected from the 180 fruit per treatment group.

### Measurement of weight loss and color

Weight loss (%) was determined by comparing fruit weight at 0 d to that on each sampling day. Color parameters (L^*^, a^*^, b^*^, and hue angle) were measured using a Konica Minolta Chroma meter CR400 (Japan). Chroma (C) was calculated as $\sqrt{{{\rm{a}}}^{2}+{{\rm{b}}}^{2}}$. Whiteness index (WI), color index (CI), and browning index (BI) were computed following the previous study^5^.

### Soluble protein, MDA, H_2_O_2_, and total antioxidant capacity

Soluble protein was quantified by the Coomassie Brilliant Blue G-250 assay. Malondialdehyde (MDA), hydrogen peroxide (H_2_O_2_) and total antioxidant capacity were measured according to Zhou et al.^4^.

### SOD, POD, CAT, and APX activities

Superoxide dismutase (SOD; EC 1.15.1.1) activity was determined by the inhibition of nitro-blue tetrazolium photoreduction. Peroxidase (POD; EC 1.11.1.7) activity, catalase (CAT; EC 1.11.1.6), and ascorbate peroxidase (APX; EC 1.11.1.11) was measured according to Zhou et al.^4^.

### Chlorophyll content and metabolism-related enzymes

Chlorophyll content was determined spectrophotometrically, with chlorophyll a and b calculated using Liu et al.^11^. Activities of key chlorophyll degradation enzymes, chlorophyllase (CLH), pheophytinase (PPH), magnesium-dechelatase (MDCase), pheophorbide a oxygenase (PAO), red chlorophyll catabolite reductase (RCCR), and 7-hydroxymethyl chlorophyll a reductase (HCAR) were assayed as described by previous study^13,17,23,27,33,34^.

### RNA extraction, library construction, sequencing, and annotation

Total RNA was isolated from fruit tissues using TRIzol® (Invitrogen, USA) and quantified spectrophotometrically using the ND-2000 (NanoDrop Thermo Scientific, Wilmington, DE, USA). RNA quality was assessed by measuring the A260/A280 ratio (1.8–2.2), A260/A230 ratio (≥2.0), RNA Integrity Number (RIN ≥ 8.0), and 28S:18S rRNA ratio (≥1.0). Only high-quality RNA samples (>1 μg) were used for sequencing library construction. Libraries were prepared with the Illumina TruSeq^TM^ RNA Sample Preparation Kit (Illumina, USA) by enriching poly(A) mRNA, synthesizing double-stranded cDNA, and performing end-repair before PCR amplification. A total of 15 RNA-seq libraries, including fresh harvest (FH), control, SNP, MT, and SNP + MT-treated fruit after 4 d of storage, were sequenced on an Illumina NovaSeq 6000. Raw data were deposited in the NCBI database (accession number PRJNA1250921). Clean reads were assembled using Trinity. Unigenes were annotated by BLASTX searches (*E*-value < 1.0 × 10^−5^) against NR, COG, KEGG, and NCBI databases. Gene Ontology (GO) terms were assigned via BLAST2GO, and KEGG analysis was used to identify relevant metabolic pathways.

### Identification of differentially expressed genes and functional enrichment

Transcript abundance was normalized as fragments per kilobase of transcript per million mapped reads (FPKM), and gene-level FPKM was computed with RSEM. Differentially expressed genes (DEGs) were identified via EdgeR using |log_2_FC | >1 and *Q*-value ≤ 0.05. GO and KEGG enrichment analyses were considered significant at a Bonferroni-corrected *P* ≤ 0.05.

### Network analysis

A gene co-expression network was constructed using WGCNA (v1.68) for 1927 genes related to antioxidant capacity, chlorophyll metabolism, transcription factors, and protein kinases based on selected gene expression data. After correcting for batch effects, a signed network was generated (soft-thresholding power *β* = 9) based on topological overlap. Modules were identified with the Dynamic Tree Cut algorithm, setting a minimum module size of 30 and a merge cut height of 0.25. The module eigen-gene (ME) was derived to investigate associations with antioxidant and chlorophyll metabolic processes. Gene significance (GS), module membership (MM), and intra-modular connectivity (Kin) were used to rank genes. Modules (Blue, Yellow, Brown, Turquoise) most strongly linked to antioxidant and chlorophyll pathways were visualized in Cytoscape by selecting the top 30 genes and edges with weight ≥0.02.

### Quantitative real-time PCR analysis

Real-time PCR was performed following the method described by Zhou et al.^4^. Primers were designed using Primer Premier version 5.0 (Supplementary Table 1), yielding amplicons between 73 and 232 bp. First-strand cDNA was synthesized from 2 µg of RNA, purified using the RNeasy Mini Kit (Qiagen, Germany), with the RevertAid™ First-Strand cDNA Synthesis Kit (Fermentas, USA). Quantitative real-time PCR was carried out on an iCycler iQ platform fitted with a multicolor real-time detection module (Bio-Rad, USA). Each 10 µL reaction mixture contained 2 µL of diluted cDNA template, 5 µL of 2xqPCRmix, 0.25 µL of each forward and reverse primer, and 2.5 µL ddH_2_O. The thermal program began with an initial denaturation at 95 °C for 30 s, followed by 40 cycles of 95 °C for 10 s, 60 °C for 30 s, and 95 °C for 15 s. A melting curve was performed from 60 °C to 95 °C with an increment of 0.5 °C, detecting the temperature at each step, and ending with a final step at 95 °C for 15 s. Transcript levels were normalized to the okra *actin* reference gene, and relative expression was calculated using the 2^−ΔΔCt^ method.

### Statistical analysis

All data were analyzed using SPSS (v19.0). Tukey’s test determined significant differences among treatment means (*P* < 0.05). Different lowercase letters above bars in figures indicate statistically significant differences.

## Supplementary information

Supplementary Information

Supplementary Data 1

Supplementary Data 2

Supplementary Data 3

Supplementary Data 4

Supplementary Data 5

## Data Availability

The raw transcriptome sequencing data generated in this study have been deposited in the NCBI Sequence Read Archive (SRA) under BioProject accession number [PRJNA1250921]. All other data supporting the conclusions of this article are included within the article and its supplementary information files. Additional datasets used and/or analyzed during the current study are available from the corresponding authors on reasonable request.

## Code availability

The bioinformatics pipelines and R scripts used for transcriptome data analysis, differential gene expression analysis, and WGCNA are available from the corresponding authors upon reasonable request. Standard software packages and their versions used in this study are detailed in the Materials and Methods section.
