# Supplementary material for: Mechanisms of SNP and melatonin effects on antioxidant and chlorophyll metabolism in postharvest okra
Source: NPJ Sci Food. 2025 Aug 25;9:188. doi: 10.1038/s41538-025-00553-1 (PMC12378788; doi:10.1038/s41538-025-00553-1)
Supplement: Supplementary file 1 — Supplementary Information [file 41538_2025_553_MOESM1_ESM.pdf]

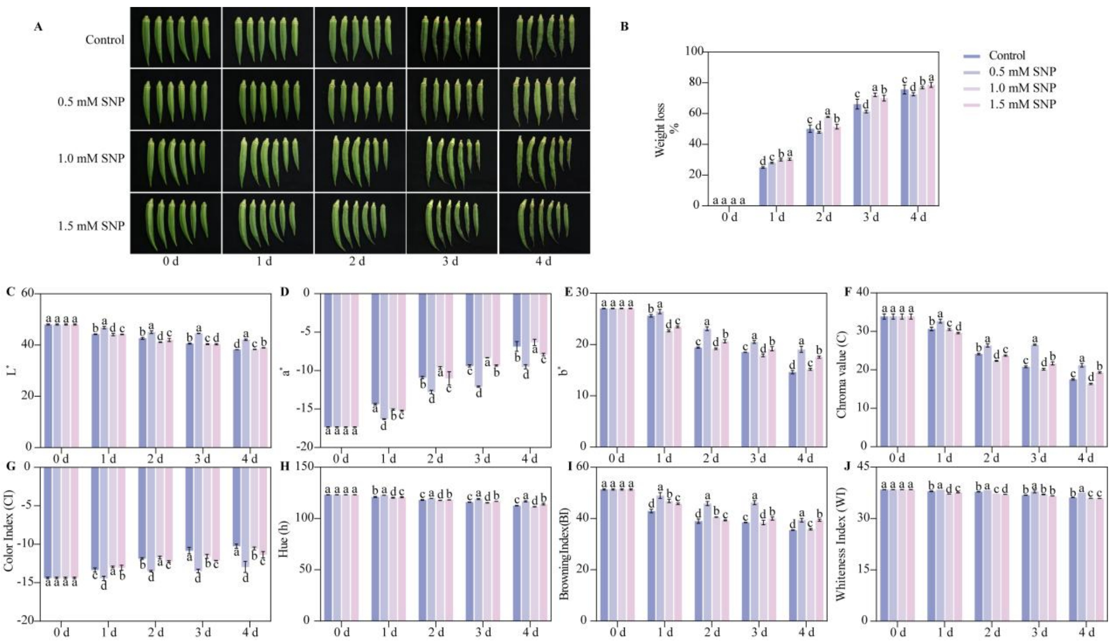

Supplementary Figure 1. Effects of different concentrations of SNP on phenotype (A), weight loss (B), L* (lightness) value (C), a* (greenness; positive values) (D), b* (yellowness) (E), chroma (F), color index (positive values) (G), h (hue angle; positive values) (H), browning index (I), and whiteness index (J) in okra fruits after 4 d of storage. Error bars represent SD (n = 3). Bars with different letters within a sampling date are significantly different (*P* < 0.05).

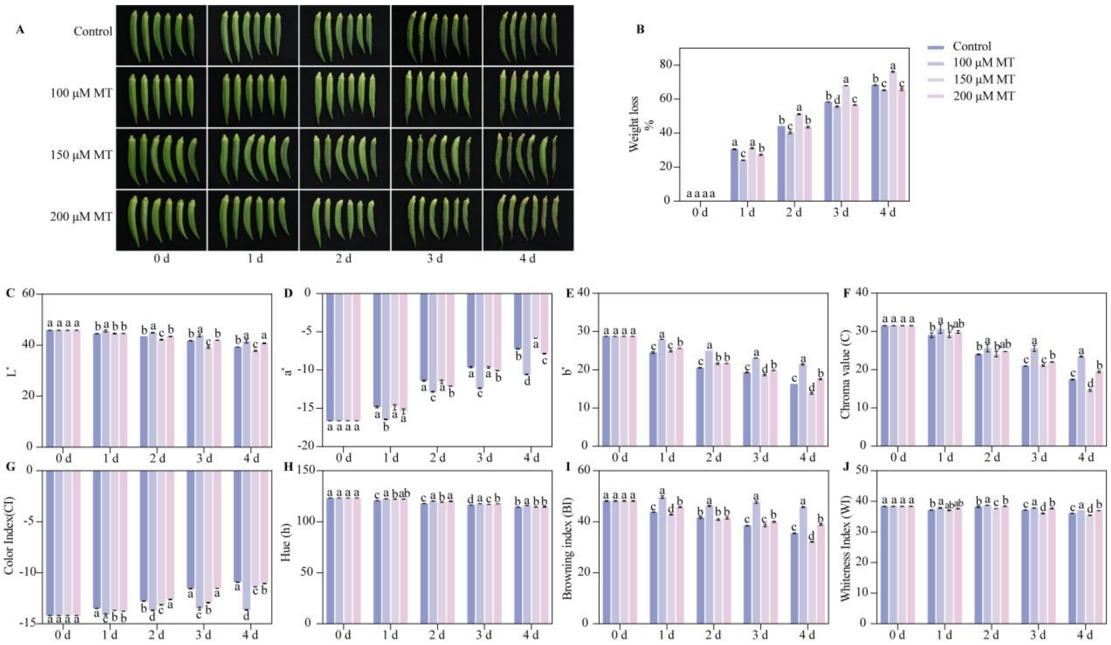

Supplementary Figure 2. Effects of different concentrations of MT on phenotype (A), weight loss (B), L* (lightness) value (C), a* (greenness; positive values) (D), b* (yellowness) (E), chroma (F), color index (positive values) (G), h (hue angle; positive values) (H), browning index (I), and whiteness index (J) in okra fruits after 4 d of storage. Error bars represent SD (n = 3). Bars with different letters within a sampling date are significantly different (*P* < 0.05).

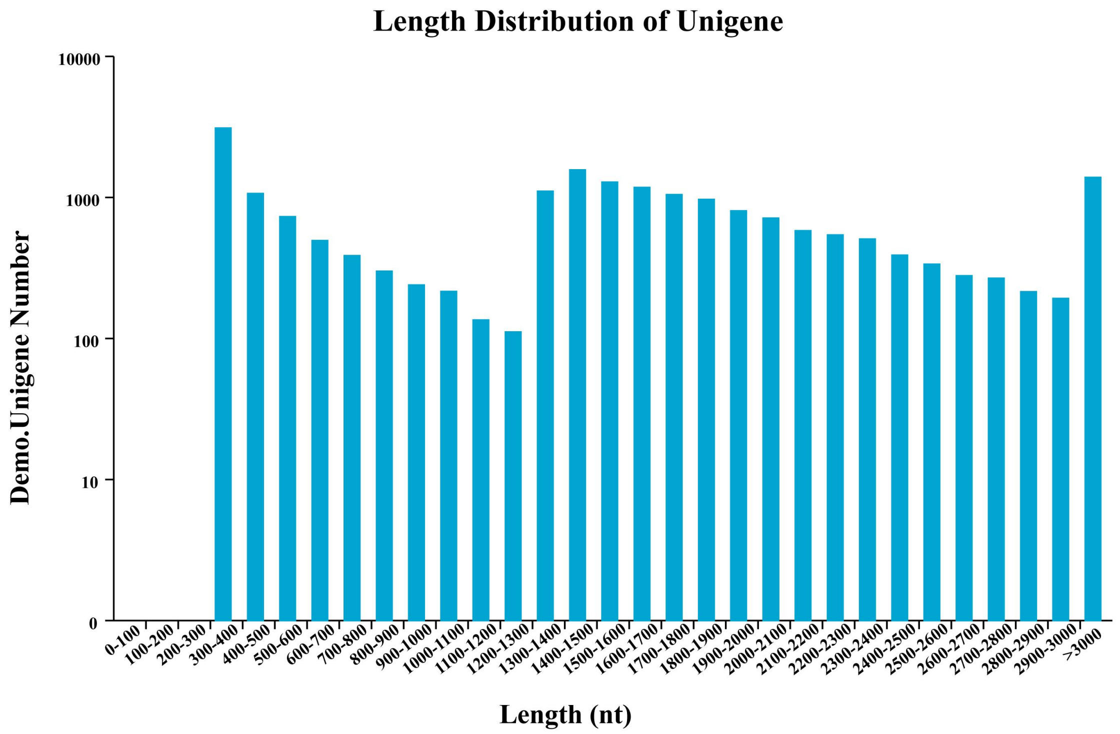

Supplementary Figure 3. Length distribution of unigenes.

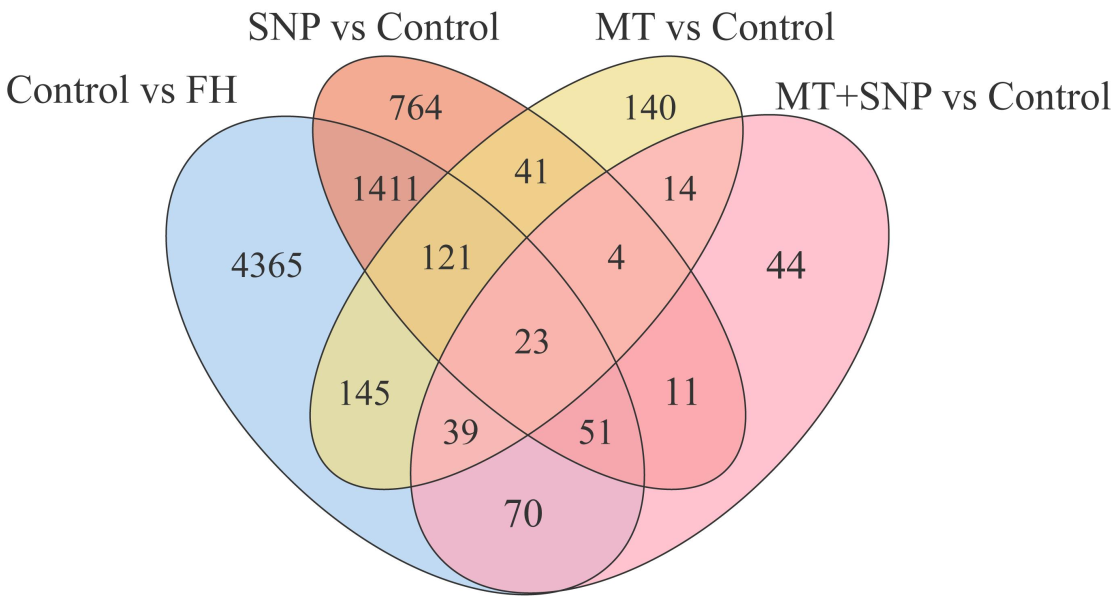

Supplementary Figure 4. Venn diagrams depicting differentially expressed genes between fresh harvest (FH) and four treatments at the end of storage.

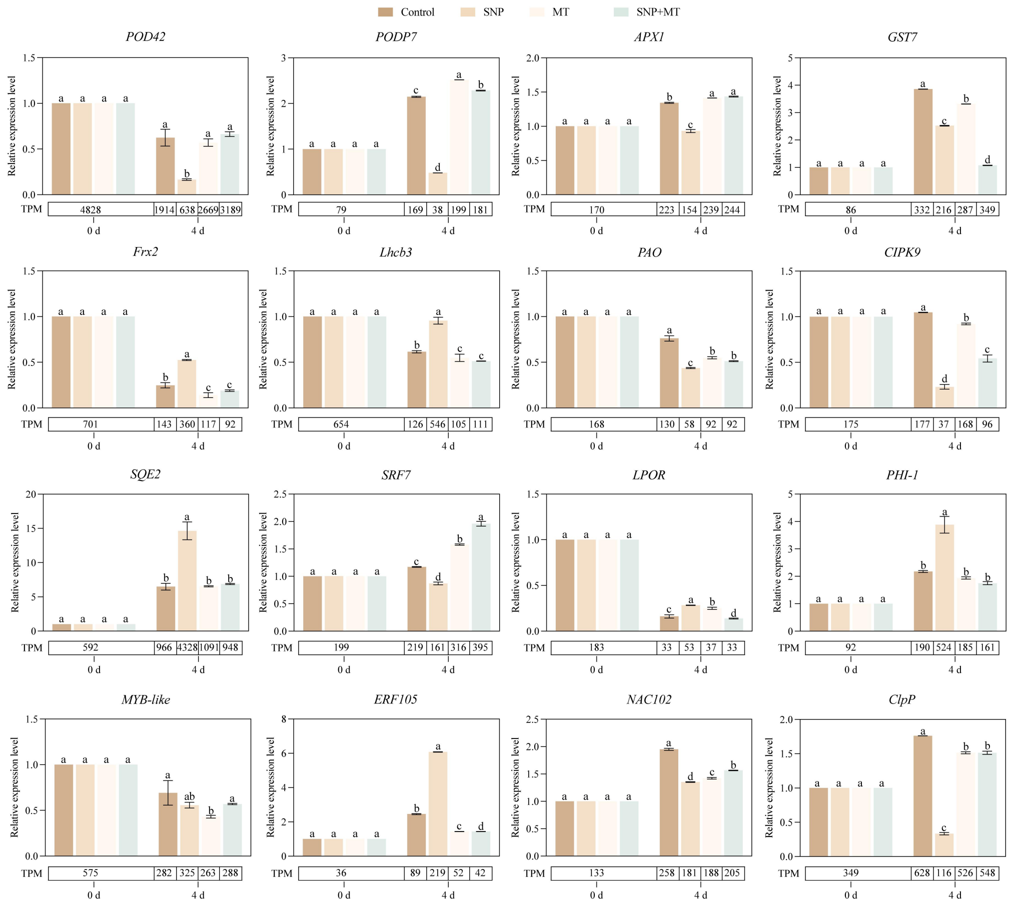

Supplementary Figure 5. Comparison of relative gene expression by RNA-seq and qRT-PCR.

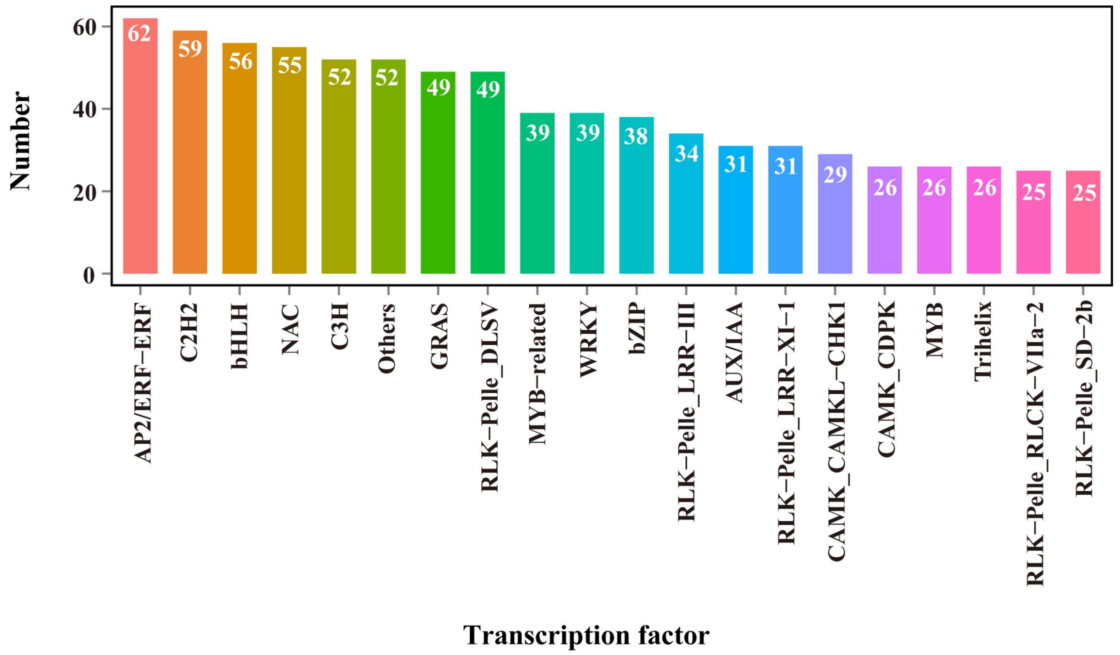

Supplementary Figure 6. The number of transcription factors and protein kinases.

Supplementary Table 1. The primer sequences used for DEGs in qRT-PCR analysis.

Gene Name	Primer sequence(5'-3')	Product size (bp)
Actin	FP:TGGCATCACTCAGCACCT	73
	RP:ACAATGGATGGACCCGAC	73
PODP7	FP:CGATTCGGGTTAGCGTTTT	151
	RP:CGGTTAGCTCGGCCATTA	151
POD42	FP:GTCAAGAACGGCGGAGAT	201
	RP: AAAGAAGCTGTAGAAAGGGAGT	201
APX1	FP:TGGGAAGGTGCCACAAGG	167
	RP:CAATGGACGGAAAACAGG	167
GST7	FP:CTTTGCCACAATAGTCACCTC	208
	RP:CGACACCCTTTATCTCATCC	208
Frx2	FP:CAGGCTCTGTTCGGGCTAA	141
	RP:AGATTCCTCGGCTGCGTC	141
LPOR	FP:ACGACGGAATCTCCATAACA	148
	RP:GCAAGAAAGCAAGGAAAAGG	148
ClpP	FP:GTGAATGAAGACGGGATGC	176
	RP:TGGCTCTGAAACTACTCGGT	176
ERF105	FP:GACAATCTACTCTAACCCAACG	195
	RP:CAAAGGTCCCAAGCCAAA	195
SQE2	FP:ATGTAGGGGACGGAGCAA	142
	RP:TCCAAGAGCATAGGCAAGA	142
PHI-1	FP:AAACAAACAAATCGTTCAGC	193
	RP:ACCGACCCAAATGTAGGC	193
SRF7	FP:TCGTGGAGTTGAGGAGTAGC	185
	RP:GATGATGTGGGGTCTGGAT	185
CIPK9	FP:GGTCCTGCGGAGTTATTCT	232
	RP:GGTGGCTTGTACCCTTTCT	232
NAC102	FP:CCGCCGAGGTTCAAAGCA	220
	RP:TCAATCCCAACGCCCCAC	220
Lhcb3	FP:AGCCTTTACCTTCGTTAGTGT	73
	RP:TCTTATTTCCTGCCCTGTTT	73
PAO	FP:ATGAGAATGGACAGGAAAGAG	150
	RP:TGTGGGAAGGATCGGAGA	150
MYB-like	FP:CTGTTGGCTTGACCACTTC	101
	RP:TGCTTACTTCCCACCATTCT	101

Supplementary Table 2. Quality control of sequencing data.

Treatment	ReadSum	BaseSum	GC(%)	N(%)	Q20(%)	CycleQ20(%)	Q30(%)
FH_1	48440389	14532116700	44.43	0.01	98.53	100	96.06
FH_2	21922569	6576770700	45.18	0.01	98.78	100	96.61
FH_3	34650961	10395288300	44.37	0.01	98.64	100	96.16
Control_1	24667056	7400116800	44.38	0	98.65	100	96.17
Control_2	30731873	9219561900	44.52	0.01	98.63	100	96.17
Control_3	20940776	6282232800	44.64	0.02	98.85	100	96.57
MT_1	33437144	10031143200	44.41	0.02	98.55	100	96.22
MT_2	25033947	7510184100	44.17	0.01	98.65	100	96.19
MT_3	41627554	12488266200	44.29	0.01	98.68	100	96.23
SNP_1	22544717	6763415100	45.18	0.01	98.74	100	96.33
SNP_2	20026068	6007820400	44.57	0.03	98.59	100	96.1
SNP_3	21365260	6409578000	44.6	0.02	99.04	100	97
MT+SNP_1	21849739	6554921700	45.24	0.07	98.45	100	95.84
MT+SNP_2	39376361	11812908300	44.54	0.02	98.99	100	96.94
MT+SNP_3	22478584	6743575200	44.59	0.01	98.49	100	95.76

Supplementary Table 3. Statistical table of assembly results.

Length Range	Transcript	Unigene
300-500	5893	4163
501-1000	4580	2147
1001-2000	31927	8402
2000+	17727	5399
Total Number	60127	20111
Total Length	104801304	31370312
N50 Length	1928	1948
Mean Length	1743.00	1559.86

Supplementary Table 4. Functional annotation analysis.

Annotation Database	Annotated Number	300<=length<1000 bp	length>=1000 bp
COG	6167	1316	4851
GO	15436	4416	11020
KEGG	13693	3709	9984
KOG	11563	3253	8310
Pfam	14877	3367	11510
Swissprot	14541	4095	10446
TrEMBL	18907	5414	13493
eggNOG	16540	4631	11909
NR	18954	5444	13510
All	19097	5522	13575

Supplementary Table 5. Up- and downregulated treatment-related DEGs in okra fruit after 4 d of storage.

Comparison group	Up	Down	Total
Control vs FH	3288	2937	6225
SNP vs Control	1187	1239	2426
MT vs Control	262	265	527
SNP+MT vs Control	125	131	256
Postharvest senescence-specific DEGs	2190	2175	4365
SNP-specific DEGs	451	313	754
MT-specific DEGs	75	65	140
SNP+MT-specific DEGs	31	13	44

Supplementary Data 1. GO terms significantly enriched in the specific DEGs of okra fruit in Control vs. FH (A), MT vs. Control (B), SNP vs. Control (C), and MT+SNP vs. Control (D) comparison groups.

Supplementary Data 2. KEGG analysis of specific DEGs of okra fruit in Control vs. FH (A), MT vs. Control (B), SNP vs. Control (C), and MT+SNP vs. Control (D) comparison groups.

Supplementary Data 3. Specific DEG-related TFs of okra fruit in Control vs. FH (A), MT vs. Control (B), SNP vs. Control (C), and MT+SNP vs. Control (D) comparison groups.

Supplementary Data 4. Correlation between four modules and physiological indices.

Supplementary Data 5. Details of 30 genes in the network diagram of four modules.
